# Changes in prescribing patterns and access to immune checkpoint inhibitors in german lung cancer patients – a claims data analysis

**DOI:** 10.1186/s12889-025-23846-2

**Published:** 2025-07-30

**Authors:** Julia Walter, Blerina Resuli, Laura Sellmer, Diego Kauffmann-Guerrero, Toki Bolt, Chukwuka Eze, Jürgen Behr, Amanda Tufman

**Affiliations:** 1 Department of Medicine V, University Hospital, LMU Munich, Munich, Germany; 2 Comprehensive Pneumology Center Munich (CPC-M), German Center for Lung Research (DZL), Munich, Germany; 3 Department of Radiation Oncology, LMU University Hospital, LMU Munich, Munich, Germany; 4 Department of Medicine V Thoracic Oncology Centre University Hospital, LMU Munich, Ziemssenstraße 1, 80336 Munich, Germany

**Keywords:** NSCLC, Secondary data, Immunotherapy, Regional differences, Viral infections, Innovation.

## Abstract

**Background:**

Recently, immune checkpoint inhibitors (ICIs) have driven profound changes in the treatment of non-small cell lung cancer (NSCLC). Their rapid integration into clinical routine is crucial for patient outcomes. However, prescribing patterns may not change immediately after authorization. Therefore, in this study we investigated factors associated with the adoption of ICI therapy for patients with advanced lung cancer in Germany following the initial regulatory approval.

**Methods:**

In this study we used German health insurance claims of 36,727 lung cancer patients diagnosed in 2015–2016. We included pre-treated patients with advanced disease. Factors potentially influencing the adoption of ICI therapies were analyzed, including demographics, residence type, hospital size, comorbidities, and metastasis location. Changes in prescribing patterns for ICI therapies were evaluated over three years using population-at-risk calculations with statistical analysis conducted using techniques including multivariate Cox regression.

**Results:**

Overall, we identified 9,726 pre-treated patients with advanced lung cancer in our dataset. Of these, 285 received ICI therapy during the course of the disease. These initial patients receiving ICI therapy were significantly younger and were more often treated in bigger hospitals. At first, uptake of ICI therapy was slow but started to increase from 1.1% in 01/2017 to 8.6% in 12/2019. Multivariate Cox regression showed that being treated in a bigger hospital (HR = 1.49, *p* = 0.001), having M1a vs. M1b or c metastases (HR = 2.65, *p* < 0.0001), being diagnosed in 2016 vs. 2015 (HR = 3.39, *p* < 0.0001), and having a comorbidity of COPD (HR = 1.46, *p* = 0.004), led to higher, faster adoption of ICI therapy.

**Conclusion:**

Introducing novel therapies necessitates a deliberate focus on disseminating information and enhancing accessibility across healthcare facilities of varying sizes.

## Background

Over the past decade, the treatment landscape for advanced or metastatic non-small cell lung cancer (NSCLC) has undergone a profound transformation. Central to this shift has been the advent of immune checkpoint inhibitors (ICIs), particularly those targeting the PD-1/PD-L1 pathway. Initial clinical trials demonstrated that ICIs offered clinical benefit in the second-line setting, leading to several regulatory approvals.

The initial checkpoint inhibitor studies showed a benefit in the second line setting, and in March and June 2015 nivolumab received approval from the US Food and Drug Administration (FDA) and the European Commission (EC) respectively, for second-line treatment of advanced squamous NSCLC, regardless of PD-L1 expression levels on tumor cells [1]. Nivolumab was subsequently approved for second-line treatment of advanced non-squamous NSCLC in the US (October 2015) and the European Union (April 2016) regardless of PD-L1 expression conferring improved efficacy compared to docetaxel. In July 2015 and October 2015, the FDA and the EC, granted approval for pembrolizumab as second-line treatment for patients with PD-L1 positive (≥ 1%) advanced NSCLC [2–5]. Atezolizumab received approval from the FDA and the EC in October 2016 and September 2017, respectively, for second-line treatment of advanced NSCLC regardless of PD-L1 status [6, 7].

In Germany, ICIs were reimbursed immediately following their approval by the European Medicines Agency (EMA), rapidly altering the standard of care for patients with advanced NSCLC who had progressed on platinum-based chemotherapy. These therapies significantly improved progression-free survival (PFS) and overall survival (OS). Consequently, timely diffusion and adoption of such therapies are critical—not only for pharmaceutical stakeholders but, more importantly, for patient outcomes. Delays or barriers in the uptake of innovative therapies, particularly in oncology, may result in substantial losses in life expectancy [8]. Previous studies have identified various factors influencing the diffusion and early adoption of novel therapies. A systematic review by Lublóy highlighted that at the prescriber level, factors such as specialization in relevant therapeutic areas, participation in clinical trials, and higher prescription volume were associated with earlier adoption [9]. At the patient level, characteristics such as younger age, higher socioeconomic status, and poorer health were linked to earlier access to new treatments. Additionally, regional disparities have been observed in the diffusion of ICIs.

Specific concerns have also shaped the early adoption of ICIs, particularly in patients with certain comorbidities. Patients with chronic viral infections, such as HIV/AIDS or hepatitis, were excluded from early clinical trials due to uncertainties regarding immune-related toxicities, viral reactivation, and therapeutic efficacy [10, 11]. This initial lack of data may have hindered early use of ICIs in these populations. Furthermore, concerns about immune-related adverse events (irAEs), particularly pneumonitis, have impacted prescribing decisions. Pulmonary comorbidities, such as asthma and interstitial lung disease (ILD), have been associated with an increased risk of pneumonitis [12, 13], potentially leading to more cautious use of ICIs in these patients during the early adoption phase. Conversely, evidence has shown that patients with chronic obstructive pulmonary disease (COPD), a common comorbidity in lung cancer, respond well to ICI therapy without an increased risk of pneumonitis [14, 15].

Therefore, in this study, we aimed to analyze differences in the initial changes in prescribing patterns and diffusion of ICI therapies in lung cancer in Germany, with a focus on hospital volume, urban and rural residency, sex, age, and specific comorbidities.

Therefore, in this study we aimed to analyze differences in the initial changes in prescribing patterns and diffusion of ICI therapies in lung cancer in Germany, with a focus on hospital volume, urban and rural residency, sex, age and certain comorbidities.

## Methods

### Data set and sample selection

We analyzed anonymized health insurance claims of 36,727 patients with incident lung cancer in 2015 or 2016, provided by the AOK Research Institute (WIdO, Berlin), covering about 30% of the German population [16]. We only included patients with stage IV (distant metastases) disease at the time of diagnosis. Additionally, we only included patients previously treated with chemotherapy, as ICI therapy was only available for pretreated patients at the time of our study period. As stage itself is not available in German claims data, we used ICD codes for secondary malignancies documented in the three months after the initial diagnosis date to identify patients with metastases in the lung (M1a), or distant metastases (M1b, M1c).

The dataset included basic patient information. Additionally, we used claims for hospitalization, outpatient hospital care, outpatient doctor visits and medications for a period of three years after diagnosis. These included German International Classification of Diseases (ICD-10-GM) codes, OPS codes (German Version of the International Classification of Procedures in Medicine), billing codes (GONR) and Anatomical Therapeutic Chemical (ATC) codes.

We identified our study sample according to a three-step process. First, we selected all patients with a diagnosis of lung cancer (ICD - C34) in 2015 and 2016. In a second step, to avoid false positives, we only included patients with at least one inpatient principal diagnosis or at least two confirmed outpatient diagnoses in two distinct quarters in the year 2015 or 2016. Third, we excluded all patients with a history of lung cancer or lung metastases in the two years prior to the diagnosis.

This study was carried out in accordance with Good Practice of Secondary Data Analysis guidelines [17]. The ethics committee of the Ludwig-Alexander university Munich approved the conduction of this study without requiring informed consent due to the retrospective, anonymized nature of the dataset.

### Identifying patients with ICI therapy

We identified patients receiving ICI therapy using OPS codes for ICI therapy in a hospital and ATC codes in an outpatient setting. ICI therapy included therapy with nivolumab, pembrolizumab, and atezolizumab.

### Patient characteristics and factors studied for association with ICI therapy

Basic data contained year of birth, sex, and regional type of residence of the patient (major city, urban, rural, remote rural). We calculated age at diagnosis using year of birth and date of diagnosis. We used the hospital ID to identify the hospital each patient was treated in, and categorized hospital size based on the number of lung cancer patients treated over two years, comparing 200 and more treated lung cancer patients and fewer than 200 treated patients. Comorbidities were derived according to Charlson comorbidity index using all inpatient, and all confirmed outpatient ICD diagnoses in the two years prior to the initial lung cancer diagnosis. As we were interested in some comorbidities of special interest ICD codes for these comorbidities were excluded in the calculation of the Charlson comorbidity score. Additionally, we further identified a few pulmonary comorbidities of special interest as well as comorbidities caused by viral infections which we expected to influence the decision for therapy with ICI. Lastly, we categorized patients according to whether they had cerebral, bone, and hepatic metastases in order to assess their effect on the decision for ICI therapy.

### Assessment of diffusion over time

In order to assess the diffusion of ICI therapy over time we calculated the population at risk for each month of the three-year observation period. The population at risk in each month was calculated as the cumulative number of patients diagnosed with lung cancer minus the cumulative number of deaths that occurred until this time. In the analysis of specific aspects e.g. hospital size, the population at risk was calculated for each category separately. Based on the population at risk we then calculated the proportion of patients with ICI therapy at each point in time, using the cumulative number of patients with ICI therapy minus the number of deceased patients with ICI therapy.

### Statistical analysis

Patient characteristics were presented as absolute and relative frequencies and means with standard deviation, and compared using Student’s t-test and Chi^2^-test, respectively. Multivariate Cox regression analysis was used to identify factors significantly associated with time until ICI therapy. Proportional hazard assumption was assessed using Schoenfeld residuals. A significance threshold of alpha < 0.05 was applied for all analyses. All analyses were performed using R Studio with R version 4.0; tables and figures were created in Microsoft Excel and R Studio [18].

## Results

### Patient characteristics

In total, we identified 9,726 pre-treated patients with stage IV lung cancer in our dataset. Of these, 285 (2.9%) received ICI therapy during the course of the disease. Patients receiving ICI therapy were significantly younger (63.4 vs. 65.8 years, *p* < 0.0001), and more frequently had stage IV M1a (24.9% vs. 15.1%, *p* < 0.0001) and less frequently M1b/M1c (75.1% vs. 84.9%) compared to patients that did not receive ICI therapy. Of all patients with ICI therapy 59.6% were treated in hospitals with a size of 200 and more treated lung cancer patients, which was significantly higher than the 40.8% patients without ICI therapy (*p* < 0.0001). Table 1 displays further patient characteristics stratified by ICI therapy, and the distribution of comorbidities and metastasis location across the two groups.

**Table 1:** Patient characteristics of lung cancer patients stratified by therapy with immune checkpoint inhibitors

	ICI therapy (*n* = 285)		no ICI therapy (*n* = 9 441)		*p*-value
	mean	sd	mean	sd	
**age**	63.4	9.5	65.8	9.5	< 0.0001
**CCI score**	2.4	1.97	2.4	2.01	0.88
	n	%	n	%	p-value
year of diagnosis					
2015	67	23.5%	4794	50.8%	
2016	218	76.5%	4647	49.2%	< 0.0001
sex					
male	184	64.6%	6173	65.4%	
female	101	35.4%	3268	34.6%	0.82
age group					
< 65	152	53.3%	4173	44.2%	
65 to 74	93	32.6%	3255	34.5%	
> 74	40	14.0%	2013	21.3%	0.002
M category					
M1a	71	24.9%	1425	15.1%	
M1b, M1c	214	75.1%	8016	84.9%	< 0.0001
regional type of residence					
major city	71	24.9%	2682	28.4%	
urban	85	29.8%	3310	35.1%	
rural	65	22.8%	1794	19.0%	
remote rural	62	21.8%	1626	17.2%	0.03
hospital size (# of LC patients)					
200 patients and more	170	59.6%	3849	40.8%	
Fewer than 200 patients	115	40.4%	5592	59.2%	< 0.0001
comorbidities					
diabetes	92	32.3%	2862	30.3%	0.52
hepatitis	1	0.4%	108	1.1%	0.38
HIV/AIDS	0	0.0%	18	0.2%	1.00
chronic bronchitis	98	34.4%	2615	27.7%	0.02
emphysema	42	14.7%	971	10.3%	0.02
COPD	144	50.5%	3860	40.9%	0.001
asthma	44	15.4%	1129	12.0%	0.09
bronchiectasis	3	1.1%	53	0.6%	0.23
interstitial lung diseases	8	2.8%	231	2.4%	0.85
metastases location					
brain metastases	90	31.6%	2453	26.0%	0.04
bone metastases	137	48.1%	3283	34.8%	< 0.0001
hepatic metastases	97	34.0%	2946	31.2%	0.34

Comparison of patient characteristics of patients with lung cancer with and without therapy with immune checkpoint inhibitors. Age and CCI score are presented as means with standard deviation and categorical variables as absolute and relative frequencies. Comparison of means with Students’ t-test and Chi^2^-test for categorical variables. Hospital size was calculated using the number of patients with tumor directed therapy in each hospital

*ICI* immune checkpoint inhibitor, *CCI* Charlson cormorbidity score, *LC* lung cancer

### Deviations from the overall proportion of ICI therapy

**Fig. 1:**
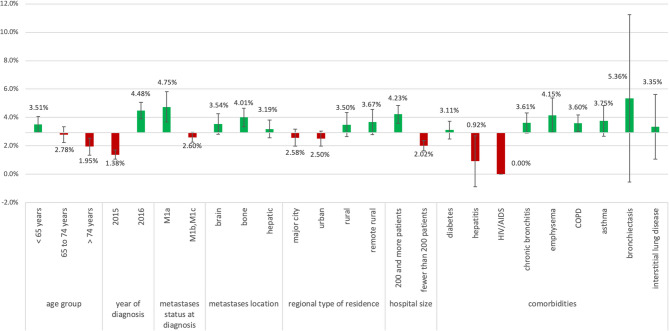
Deviations from overall proportion of patients with therapy with ICI depending on specific patient characteristics

Figure 1 shows that patients diagnosed in 2015 were less likely whereas patients diagnosed in 2016 were more likely to receive ICI therapy compared to the overall proportion of 2.93%. Additionally, you can see that the proportion of patients with M1a receiving ICI, as well as patients treated in bigger hospitals was higher than in the overall population. Patients with pulmonary comorbidities like chronic bronchitis, emphysema, COPD, asthma, bronchiectasis, and even ILD received ICI therapy more often than the overall population. Only one patient with hepatitis and no patients with HIV/AIDS received ICI therapy. Patients in major cities and urban areas received ICI therapy to a lesser extent than patients with rural and remote rural residence. However, the proportion of patients treated in bigger hospitals was significantly (*p* < 0.0001) higher in major cities (50.3%), than in urban (40.3%), rural (38.2%), and remote rural areas (32.1%).

**Fig. 2:**
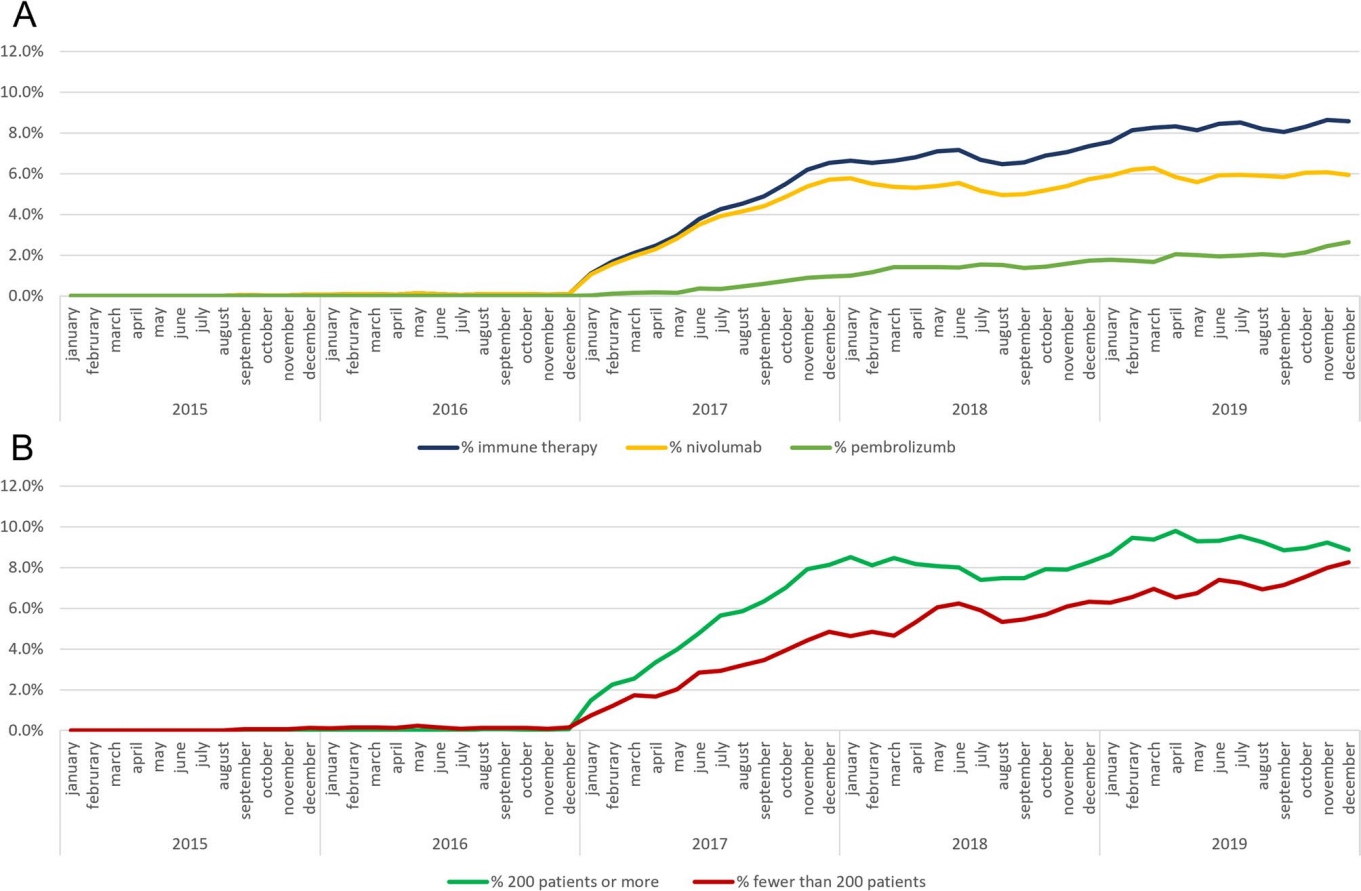
Trend in uptake of therapy with immune checkpoint inhibitors after authorization

Deviations from the overall proportion of patients receiving ICI therapy of 2.55% regarding different demographic and clinical aspects. HIV/AIDS = Human Immunodeficiency Virus/ Acquired Immunodeficiency Syndrome, COPD = Chronic obstructive pulmonary disease, ICI = Immune checkpoint inhibitor. (Figure 2)

Uptake of ICI therapy in at-risk population. Population at risk was defined monthly over the three-year observation period as the cumulative number of patients diagnosed with lung cancer minus cumulative deaths up to that time. For subgroup analyses (e.g., by hospital size), this was calculated separately for each category. The proportion of patients receiving ICI therapy at each time point was based on the cumulative number of patients who received ICI therapy, subtracting those who had died.

(A) Proportion of eligible patients per month receiving therapy with immune checkpoint inhibitors. (B) Proportion of eligible patients per month receiving therapy with immune checkpoint inhibitors stratified by hospital size of the treating hospital.

### Uptake and diffusion over time

The first ICI therapy captured by our dataset was applied in September 2015. The proportion of patients receiving ICI therapy stayed under 1% of the eligible patients until December of 2016, then it increased to over 1.1% of at-risk patients in January 2017. The highest proportion of at-risk patients received ICI therapy in November 2019 with 8.6%. Figure 2 (A) displays this trend and shows the trend in nivolumab and pembrolizumab specifically. As can be seen in Fig. 2 (B), uptake was faster in bigger hospitals compared to smaller ones. Levels of uptake in bigger hospitals remained above the ones for smaller hospitals over the whole study period. Uptake in patients with pulmonary comorbidities differed according to the specific comorbidity; however, we did not see a clear trend here.

### Median time to fist immune therapy

Table 2 displays median time to first ICI therapy depending on the factors we hypothesized to be associated. Overall, median time to first therapy was 14.1 months (CI 12.9, 15.8). We found that median time to first therapy was only significantly associated with year of incidence.

**Table 2:** Median time to first ICI therapy depending on patient characteristics

		median	CI	*p*-value
	overall	14.1	[12.9,15.8]	
age group	< 65 years	15.4	[12.8,16.9]	
	65 to 74 years	13.5	[11.3.15.8]	
	> 74 years	13.5	[11.7,17.1]	0.35
year of diagnosis	2015	24.4	[21.7,28.0]	
	2016	12.2	[10.5,13.3]	< 0.0001
sex	male	13.7	[12.3,15.6]	
	female	15.0	[13.0,18.1]	0.13
metastases status at diagnosis	M1a	15.4	[12.2,17.7]	
	M1b, M1c	14.0	[12.4,15.6]	0.70
metastases location	brain	16.7	[13.0,19.6]	0.20
	bone	13.4	[12.3,16.6]	0.80
	hepatic	13.4	[10.2,15.0]	0.20
regional type of residence	major city	16.4	[13.0,18.8]	
	urban	14.5	[10.8,16.8]	
	rural	12.7	[9.4,16.8]	
	remote rural	13.6	[12.2,18.1]	0.66
hospital size	200 and more patients	13.5	[12.3,15.4]	
	fewer than 200 patients	15.6	[12.4,18.6]	0.43
comorbidities	diabetes	14.45	[12.9,17.1]	0.90
	hepatitis	33.7	NA	NA
	HIV/AIDS	NA	NA	NA
	chronic bronchitis	15.8	[13.3,18.1]	0.20
	emphysema	15.9	[11.9,19.1]	0.30
	COPD	14.1	[12.4,12.4]	0.40
	asthma	16.1	[13.6,19.1]	0.50
	bronchiectasis	24.1	[14.0,NA]	0.30
	interstitial lung disease	18.2	[6.8,27.3]	0.70

Univariate comparison of time until first ICI therapy by certain patient characteristics. Time until first ICI therapy presented as median with Confidence Interval. P-values from LogRank test

*CI * Confidence interval, *HIV/AIDS* Human Immunodeficiency Virus/Acquired Immunodeficiency Syndrome, *COPD* Chronic obstructive pulmonary disease, *ICI* Immune checkpoint inhibitor

### Multivariate cox regression

The multivariate Cox regression showed no factors significantly associated with a lower adoption of ICI therapy. Patient characteristics with a higher and faster receipt of ICI therapy were being treated in a bigger hospital, living in a remote rural area vs. a major city, and being diagnosed in 2016 vs. 2015. Tumor characteristics and comorbidities significantly associated with higher and faster receipt were having M1a vs. M1b or M1c, and having COPD. The proportional hazards assumption was tested using Schoenfeld residuals, and no relevant violations were detected. Tables 3 and 4 display Hazard ratios with p-values and interpretations from the multivariate Cox regression model. (Tables 3 and 4)

**Table 3:** Results from Cox regression analysis of time to therapy with immune checkpoint inhibitors

	HR	coef	se	Wald	*p*-value
age in years	0.99	−0.01	0.01	−1.82	0.07
male vs. female	1.17	0.16	0.13	1.22	0.22
hospital size of 200 and more vs. fewer than 200	1.49	0.40	0.12	3.22	0.001
urban vs. major city	0.95	−0.05	0.16	−0.33	0.74
rural vs. major city	1.40	0.34	0.17	1.95	0.05
remote rural vs. major city	1.70	0.53	0.18	2.98	0.003
M1a vs. M1b, M1c	2.65	0.97	0.16	5.95	< 0.0001
incidence 2016 vs. 2015	3.39	1.22	0.14	8.49	< 0.0001
Charlson score	1.02	0.02	0.04	0.52	0.61
Diabetes vs. no diabetes	1.30	0.26	0.15	1.68	0.09
hepatitis infection vs. no hepatitis infection	0.42	−0.88	1.00	−0.87	0.38
cerebral metastases vs. no cerebral metastases	2.44	0.89	0.14	6.45	< 0.0001
bone metastases vs. no bone metastases	3.18	1.16	0.13	8.66	< 0.0001
hepatic metastases vs. no hepatic metastases	2.64	0.97	0.14	7.02	< 0.0001
chronic bronchitis vs. no cerebral metastases	1.08	0.08	0.13	0.59	0.56
Emphysema vs. no emphysema	1.31	0.27	0.18	1.54	0.12
COPD vs. no COPD	1.46	0.38	0.13	2.87	0.004
Asthma vs. no asthma	1.12	0.11	0.17	0.65	0.51
bronchiectasis vs. no bronchiectasis	1.24	0.22	0.60	0.36	0.72
interstitial lung disease vs. no interstitial lung disease	1.21	0.19	0.37	0.51	0.61

Hazard Ratios with p-values from multivariate Cox regression model of time-until first ICI therapy

*HR* Hazard ratio, *se* standard error, *COPD* chronic obstructive pulmonary disease, *ICI* Immune checkpoint inhibitor

**Table 4:** Interpretation of factor with significant association from Cox regression analysis

Factor	HR	95% CI	*p*-value	Interpretation
Hospital size (≥ 200 vs. <200)	1.49	1.15–1.93	0.001	Larger hospitals → faster therapy
Residence (remote rural vs. major city)	1.70	1.22–2.36	0.003	Remote rural → faster therapy
Residence (rural vs. major city)	1.40	1.00–1.96	0.05	Rural → faster therapy
Metastases category (M1a vs. M1b/M1c)	2.65	1.95–3.60	< 0.0001	M1a → much faster therapy
Year of diagnosis (2016 vs. 2015)	3.39	2.54–4.52	< 0.0001	Diagnosed in 2016 → faster therapy
Cerebral metastases (yes vs. no)	2.44	1.88–3.16	< 0.0001	Presence → faster therapy
Bone metastases (yes vs. no)	3.18	2.42–4.18	< 0.0001	Presence → faster therapy
Hepatic metastases (yes vs. no)	2.64	1.92–3.63	< 0.0001	Presence → faster therapy
COPD (yes vs. no)	1.46	1.12–1.91	0.004	COPD → faster therapy

Hazard Ratios with p-values from multivariate Cox regression model of time-until first ICI therapy

*HR* Hazard ratio, *COPD* chronic obstructive pulmonary disease

## Discussion

In this study, our goal was to assess the uptake of ICI therapies as second- or third-line treatment of patients with advanced lung cancer at the beginning of market authorization in Germany. The analysis was based on a comprehensive dataset, representing approximately 30% of the German population, which offering a representative view of the country’s lung cancer treatment landscape.

We found that initial uptake was slow but began to increase at the end of 2016 and the beginning of 2017. A main driver for faster and higher uptake was access to hospitals with a high volume of lung cancer patients whereas uptake was minimal in patients with hepatitis and HIV/AIDS. The location of metastases also seemed to play a role in the decision for ICI therapy favoring patients with less advanced disease.

It is not surprising that at the onset of ICI therapy market authorization in Germany, the adoption rate was slow, given the initially narrow treatment indications. However, towards the end of 2016 and early 2017, a notable increase in uptake was observed. This likely reflects the expansion of approved indications beyond squamous cell carcinoma, which enabled the inclusion of pre-treated patients with non-squamous NSCLC.Consequently, the proportion of patients receiving ICI therapy rose from 1.1% in January 2017 to 8.6% in November 2019.

Compared to other studies the overall proportion of patients treated with ICI therapy was lower [19, 20]. However, this can be explained by the nature of this dataset and the methods we used. On the one hand we only had patients diagnosed in 2015 and 2016, a period when ICI therapies had only recently been approved in Germany. Other studies, by contrast, examined a longer timeframe [19, 20]. On the other hand, our dataset does not include information on the histological subtype of the cancer, which made the calculation of the number of eligible patients more complex.

A compelling finding in our study was the impact of hospital volume on ICI therapy uptake. Larger hospitals, with higher lung cancer patient volumes, demonstrated faster and more substantial adoption of ICI therapies. This phenomenon can be attributed to the expertise of healthcare professionals at these facilities, enabling them to stay up-to-date with the latest treatment options and adopt innovations swiftly. Often, these hospitals are also academic centers, although, a study from the United States also found high levels of uptake of ICI therapy also in community-based practices [19]. Still, our analysis showed that treatment in a large hospital was associated with faster and more frequent initiation of ICI therapy. This underscores the critical role of structural healthcare factors—particularly the availability of specialized expertise and resources—in the adoption of innovative therapies.Patients with chronic viral infections, such as hepatitis and HIV/AIDS, showed minimal uptake of ICI therapy. Namely, no patient with HIV/AIDS and only one with hepatitis received ICI therapy. However this did not result in a significant difference between patients receiving ICI and patients not receiving ICI. Early clinical trials often exclude such patients due to concerns about viral reactivation, toxicity, and efficacy [10, 11]. Nevertheless, limited data from the literature indicate that newer trials have shown the safety and efficacy of ICI therapy in these populations, potentially leading to reduced disparities in the future. For example, a clinical trial of pembrolizumab and another trial of durvalumab in patients with HIV on anti-retroviral therapy and advanced-stage cancer, reported that both therapies did not impair CD4 + cell counts or viral suppression [21, 22]. Additionally, several studies have shown that toxicity and efficacy rates were similar to those observed in patients without chronic viral infections without viral reactivation [23]. However, due to the very small number of patients with chronic viral infections in our cohort, particularly those receiving ICI therapy, these findings should be interpreted with caution. The limited sample size in these subgroups precludes robust statistical analysis and may not accurately reflect real-world uptake or outcomes in broader populations. Future research with larger, targeted cohorts is needed to draw more definitive conclusions regarding treatment patterns and clinical outcomes in patients with HIV or hepatitis.

All studied respiratory diseases did not result in lower uptake of ICI therapy. In the multivariate Cox regression COPD was even significantly associated with a higher and faster diffusion of ICI therapy, while all other respiratory comorbidities were not significantly associated. This finding is especially interesting as it confirms studies which found no increased risk for irAEs in COPD patients [14] and even better survival outcomes [15]. A reason for the high proportion of COPD patients receiving ICI therapy could be that patients with a smoking history in general have a good response to ICI therapy. This may lead to clinicians more frequently considering this patient group as suitable candidates for immunotherapy. One potential explanation is that COPD patients often have a history of smoking, which is linked to higher tumor mutational burden and better ICI response.

Another important observation was the slower uptake of ICI therapy in older patients and those ineligible for phase III randomized clinical trials. Only 1.9% of patients older than 74 years received ICI therapy, compared to 3.5% of patients under the age of 65 years. This pattern aligns with previous studies, indicating a gap in accessing innovative treatments for specific patient groups [24]. Although age was associated with lower uptake in univariate comparisons, it did not remain a significant predictor in the multivariate Cox model. This suggests that other factors, such as hospital size or tumor stage, had a more substantial influence on treatment decisions.

Overall, uptake was slower in patient groups that are routinely ineligible to participate in phase III randomized clinical trials. A recent study in clinical practice demonstrated that these ineligible patients received ICI therapy at higher rates later, suggesting the need for a more inclusive approach in clinical trials and therapeutic decisions [20]. Although we did find these discrepancies, our analysis only focused on the beginning of ICI therapies. Studies suggest that nowadays especially older patients and patients who used to be too frail for therapy benefit from ICI monotherapy.

Interestingly, patients living in rural areas demonstrated higher uptake of ICI therapy compared to their urban counterparts. Although a similar trend was observed in a study from the United States [24], the reasons behind this phenomenon remain unclear. Possible explanations may include better individualized care, specific regional programs, or structural differences in healthcare delivery systems. Further research is warranted to explore the contributing factors and ensure equitable access to ICI therapies across diverse geographical regions.

Patients with less advanced disease showed a higher likelihood of receiving immune checkpoint inhibitor (ICI) therapy within our studied cohort. This phenomenon potentially signifies a certain apprehension towards implementing innovative yet economically demanding therapeutic regimens in patients burdened with extensive tumor presence. Now established clinical precedent underscores that a lower tumor burden correlates with improved patient outcomes in ICI therapy in NSCLC [25, 26]. Overall this might reflect a selective approach in clinical decision-making, where patients with lower tumor burden are preferentially offered immunotherapy due to their more favorable prognosis or anticipated lower risk of complications. Moreover, an in-depth retrospective analysis of the KEYNOTE-001 trial unveiled a distinct association between diminished tumor burden and favorable clinical response in melanoma, alongside enhanced overall survival (OS) rates, particularly evident in univariate and multivariate assessments. This underscores the independent predictive capacity of tumor burden concerning the therapeutic response to pembrolizumab, an established ICI [27].

While our study primarily reflects the early phase of immune checkpoint inhibitor (ICI) adoption in Germany, comparing these findings with data from other European healthcare systems helps to better understand regional similarities and disparities. For example, studies from France and the United Kingdom have reported somewhat parallel patterns of ICI uptake, with initially slow adoption followed by rapid increases as indications expanded and reimbursement pathways stabilized [28, 29]. In France, nationwide registry data indicated that ICI therapy became increasingly integrated into treatment algorithms for advanced non-small cell lung cancer (NSCLC) beginning in 2017, with uptake rates similar to those observed in our study’s later time frame [28]. Likewise, UK data from the National Lung Cancer Audit revealed growing use of ICIs from 2017 onward, with disparities linked to hospital volume and patient demographics comparable to our findings [29]. These comparisons highlight structural and systemic factors common across European healthcare systems influencing the diffusion of novel cancer therapies.

Additionally, our analysis focused on data up to 2016, representing the start of ICI market authorization in Germany. More recent real-world studies from 2019 onwards provide evidence of continued expansion and normalization of ICI use across various patient subgroups. For instance, post-2019 reports from European registries and observational cohorts demonstrate increasing ICI utilization in older patients, those with comorbidities, and broader histological types, reflecting more inclusive clinical practices and updated guideline recommendations [30, 31]. Incorporating these recent trends reinforces the notion that our findings capture a foundational phase in ICI adoption, with subsequent years marking maturation and broader access.

Future studies should continue to monitor how these evolving patterns impact clinical outcomes and equity in treatment access across different European regions.

There are some limitations to our study. For example, German health care claims do not include any information regarding the histological lung cancer subtype as well as PD-L1 expression of the tumor or other genetic mutations such as EGFR or ALK. of the lung cancer. Therefore, estimating the eligible patient cohort was difficult. Furthermore, important clinical characteristics such as smoking status and ECOG performance status were not available in the dataset, which may introduce information bias and limit the ability to adjust for relevant prognostic factors. However, the factors potentially associated with higher or lower uptake of ICI therapy that we studied are not associated with histological subtypes. Therefore, we believe that even though our eligible patient cohort might be too high, the relationship between uptake and these factors can be studied in our cohort. As we analyzed claims of a German statutory health insurance company, reasons for or against use of ICI therapy could not be studied in this analysis.

The strengths of this study derive from the large dataset. Data from WIdO covers around 30% of the German population and therefore our analysis is representative for the whole German population. Additionally, due to this a risk for selection bias is minimal. Further, as our dataset included information on demographic factors like the residence type and the hospitals patients were treated in, but also all types of ICD diagnoses, we were able to shed a light on various different aspects at the same time.

## Conclusion

Diffusion of ICI therapy was mainly driven by uptake in bigger hospitals, even when adjusted for other factors. Introducing novel therapies necessitates a deliberate focus on disseminating information and enhancing accessibility across healthcare facilities of varying sizes, encompassing those with lower patient volumes and resident practitioners.

Moreover, following regulatory approval, there emerges a pressing need for prompt clinical trials involving individuals ineligible for initial authorization trial participation. This urgency stems from their inherent disadvantage in rapidly adopting innovative treatments, resulting from their exclusion from the early trial stages.

## Data Availability

All data are stored in a central database at the AOK Research Institute (WIdO, Berlin). We received aggregated and anonymised data of all patients meeting the above mentioned inclusion criteria. The authors confirm that the data utilized in this study cannot be made available in the manuscript, the supplemental files, or in a public repository due to German data protection laws (‘Bundesdatenschutzgesetz’, BDSG). Therefore, they are stored on a secure drive in the senior author’s institution to facilitate replication of the results. Generally, access to data of statutory health insurance funds for research purposes is possible only under the conditions defined in German Social Law (SGB V § 287). Requests for data access can be sent as a formal proposal specifying the recipient and purpose of the data transfer to the appropriate data protection agency. Access to the data used in this study can only be provided to external parties under the conditions of the cooperation contract of this research project and after written approval by the sickness fund. For assistance in obtaining access to the data, please contact the corresponding author, Julia Walter, at Julia.Walter@med.uni-muenchen.de.
